# A Rare Case of Complete Stent Fracture, Coronary Arterial Transection, and Pseudoaneurysm Formation Induced by Repeated Stenting

**DOI:** 10.1155/2015/192853

**Published:** 2015-10-12

**Authors:** Fumiaki Nakao, Masashi Kanemoto, Jutaro Yamada, Kazuhiro Suzuki, Hidetoshi Tsuboi, Takashi Fujii

**Affiliations:** ^1^Department of Cardiology, Yamaguchi Grand Medical Center, 77 Ohsaki, Hofu, Yamaguchi 747-8511, Japan; ^2^Division of Cardiology, Department of Medicine and Clinical Science, Yamaguchi University Graduate School of Medicine, 1-1-1 Minami-kogushi, Ube, Yamaguchi 755-8505, Japan; ^3^Department of Cardiovascular Surgery, Yamaguchi Grand Medical Center, 77 Ohsaki, Hofu, Yamaguchi 747-8511, Japan

## Abstract

This report describes a rare asymptomatic case of complete stent fracture, coronary arterial transection, and pseudoaneurysm formation in response to repeated stenting. The proximal and distal ends of transected coronary artery were closed, and distal bypass was performed. Coronary arterial transection can occur in patients with repeated stenting as a long-term adverse event.

## 1. Introduction

Stent fracture after drug-eluting stent (DES) deployment is an important issue, because it is strongly associated with restenosis, target legion revascularization, and stent thrombosis [1]. A report of autopsy cases with DES deployment showed stent fracture in 29% of lesions and restenosis or stent thrombosis in 67% of cases with gapped stent fracture [2].

Stent fracture can also lead to coronary pseudoaneurysm formation, which can be life-threatening [3]. The incidence of coronary pseudoaneurysm formation after DES deployment is 0.3–4.5% [4]. Management strategies for coronary pseudoaneurysm include observation, surgical treatment and interventional treatment, such as coil embolization and deployment of a polytetrafluoroethylene- (PTFE-) covered stent [3–5].

## 2. Case Report

**Figure 1:**
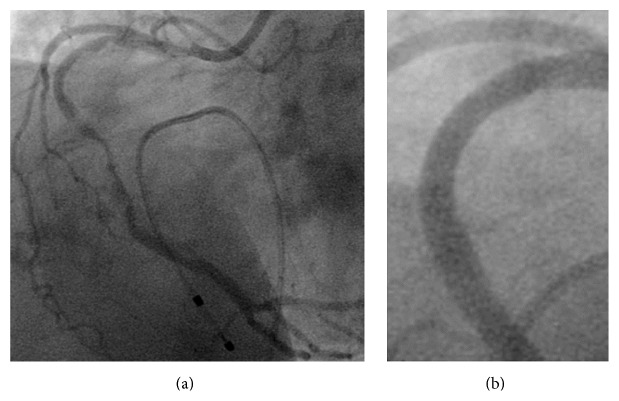
First percutaneous coronary intervention (PCI#1). (a) Baseline coronary angiography (CAG). (b) CAG after first stenting.

**Figure 2:**
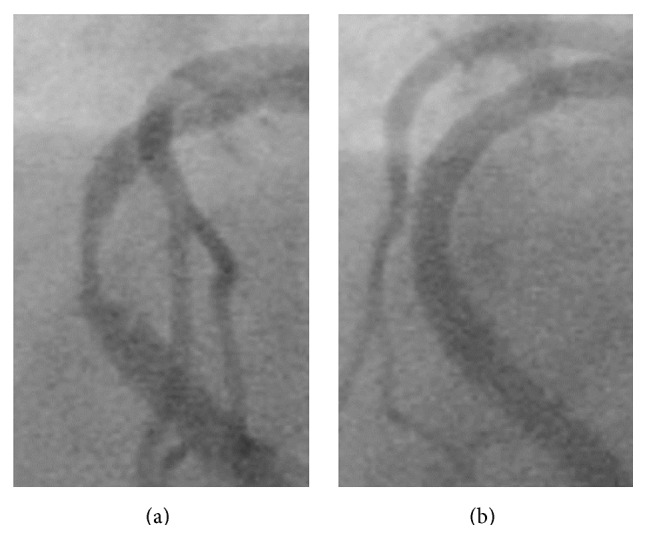
Second percutaneous coronary intervention (PCI#2). (a) Baseline coronary angiography (CAG). (b) CAG after second stenting.

**Figure 3:**
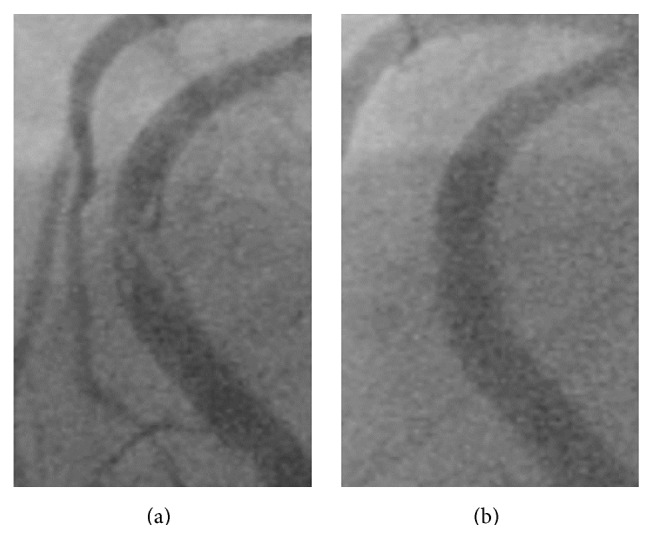
Third percutaneous coronary intervention (PCI#3). (a) Baseline coronary angiography (CAG). (b) CAG after third stenting.

**Figure 4:**
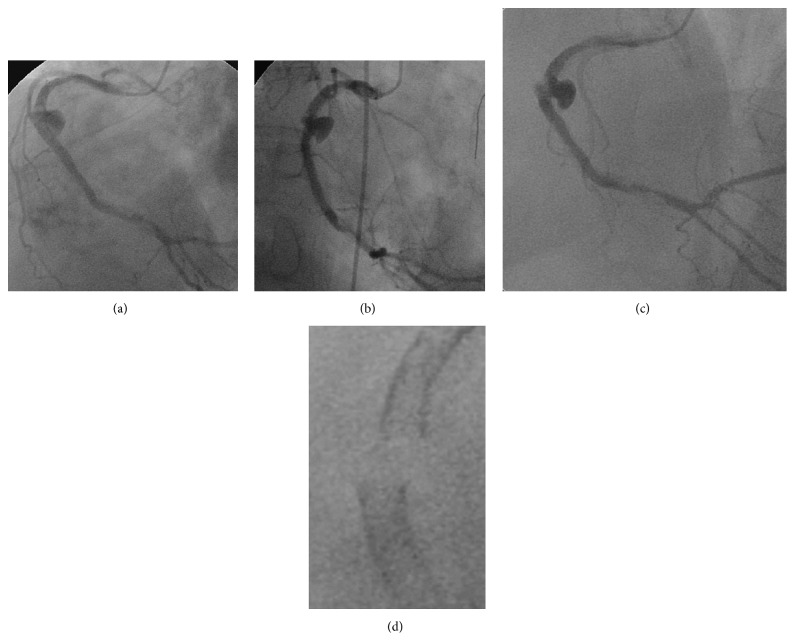
Follow-up coronary angiography showing pseudoaneurysm formation. Left anterior oblique (LAO) view (a), right anterior oblique view (b), and LAO-cranial view (c). (d) X-ray fluorography showing complete stent fracture.

A 61-year-old male undergoing chronic hemodialysis had previously underwent rotational atherectomy and stenting (TAXUS Liberte, Boston Scientific Co.) for a long, severely calcified lesion of the right coronary artery (RCA) (first percutaneous coronary intervention [PCI#1], Figure 1). Six months later, the patient underwent emergent restenting (Cypher, Cordis) for probable stent thrombosis of the mid-RCA with ST elevation (second PCI [PCI#2], Figure 2). Four months later, he underwent emergent repeat stenting (Xience V, Abbott Vascular) for probable stent thrombosis of the mid-RCA with ST elevation (third PCI [PCI#3], Figure 3). Two months later, he was admitted for follow-up coronary angiography (CAG) and was noted to be asymptomatic. CAG showed pseudoaneurysm formation in the mid-RCA (see Figures 4(a), 4(b), and 4(c) and see Clip  1 in Supplementary Material available online at http://dx.doi.org/10.1155/2015/192853), and X-ray fluorography showed complete stent fracture (Figure 4(d)). Coronary transection was suspected, because of findings of complete stent fracture and contrast media oozing all around the part of stent fracture.

## 3. Discussion

Risk factors for stent fracture include RCA stenting, long stenting, overlapped stenting, and stenting on a hinge point [6, 7]. The present patient underwent long and overlapped stenting within the RCA and therefore was at high risk for stent fracture. Drugs and polymers of DES may induce vascular inflammation and delay vascular healing [8], and they also can contribute to pseudoaneurysm formation. In this case, the vessel wall was likely exposed to a relatively high dose of DES drug and polymer (due to three overlapping stents).

**Figure 5:**
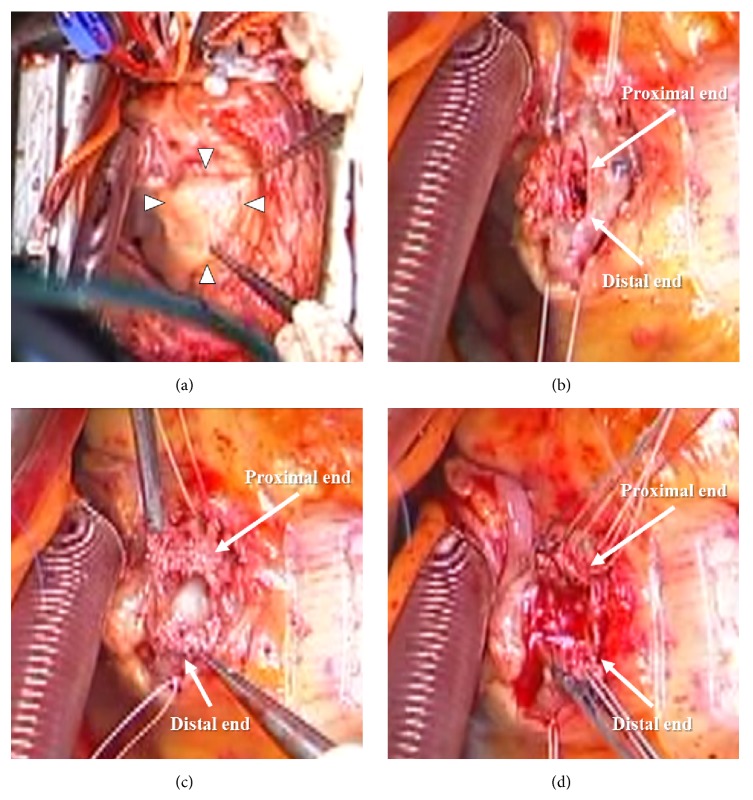
Intraoperative findings. The pseudoaneurysm is in the visceral adipose tissue (arrow heads) (a) and opened (b). (c) Coronary arterial transection. (d) Proximal and distal transected ends are closed.

Surgical treatment and a PTFE-covered stent deployment were considered for this case. However, a guidewire could perforate the wall of the pseudoaneurysm, and deployment of the PTFE-covered stent might be difficult, because previous procedures required the mother and child (4 in 6) technique. If repeated stenting for stent fracture was performed, stent fracture might occur repeatedly, leading to lethal stent thrombosis or blow-out rupture of the pseudoaneurysm. Therefore, surgical management was selected for this case. During surgery, the pseudoaneurysm was visualized in the visceral adipose tissue (arrow heads, Figure 5(a)). After the pseudoaneurysm was opened (Figure 5(b)), coronary transection was confirmed (arrows, Figure 5(b)). The proximal and distal transected ends of the mid-RCA could not be ligated because of protrusion of the overlapped fractured struts (arrows, Figure 5(c)). Therefore, proximal and distal transected ends of the mid-RCA were closed (Figure 5(d)), and distal bypass was performed.

In conclusion, this case report described a rare asymptomatic case of complete stent fracture, coronary arterial transection, and pseudoaneurysm formation in response to repeated stenting. Coronary arterial transection can occur in patients with repeated stenting as a long-term adverse event.

## Supplementary Material

Follow-up coronary angiography of right coronary artery shows pseudoaneurysm formation and complete stent fracture. Coronary transection is suspected, because of findings of complete stent fracture and contrast media oozing all around the part of stent fracture.
